# Effect of Bioactive Phytochemicals from *Phlomis viscosa* Poiret on Wound Healing

**DOI:** 10.3390/plants8120609

**Published:** 2019-12-16

**Authors:** Ludmila Yarmolinsky, Arie Budovsky, Leonid Yarmolinsky, Boris Khalfin, Vladimir Glukhman, Shimon Ben-Shabat

**Affiliations:** 1 Eastern R&D Center, Kiryat Arba 9010000, Israel; liorayarl@mail.com (L.Y.); khalphin@bgu.ac.il (B.K.); 2 Barzilai University Medical Center, Ashkelon 7830604, Israel; arieb@bmc.gov.il; 3 Arnie Miller Laboratories, Beer-Sheva 8430713, Israel; yludmila@bgu.ac.il; 4 Department of Biochemistry and Pharmacology, Faculty of Health Sciences, Ben-Gurion University of the Negev, Beer-Sheva 8410501, Israel; 5 Yarok Microbio LTD, Jerusalem 9342142, Israel; vglukhman@gmail.com

**Keywords:** Wound healing, *Phlomis viscosa*

## Abstract

*Phlomis viscosa* Poiret is an evergreen shrub growing in Israel, Turkey, Lebanon, and Syria with acknowledged pro-wound healing (WH) properties. In this study, we evaluated the pro-WH potential of selected compounds found in this plant. Among the pro-WH compounds (identified by us) was a combination of three chemicals—diosmin, 1-octen-3-ol, and himachala-2,4-diene which enhanced WH significantly both in in vitro and in vivo models. The determined phytochemicals combination could be used for the treatment of chronic wounds. The effect of the extracts, diosmin, 1-octen-3-ol on the secretion of pro-inflammatory cytokines, IL-6 (A) and IL-8 (B) by human dermal fibroblasts was significant (*p* < 0.001). In addition, the beneficial effect of extracts of *P. viscosa* and its phytochemicals on WH was evidenced by inhibiting the growth of several WH delaying microorganisms.

## 1. Introduction

The Judea region (Israel) is situated between the Judean Hills, with an elevation of up to 1000 m above sea level and a rainy Mediterranean climate, and the Dead Sea, which is the lowest place on earth with constantly warm and dry conditions [1]. These two climatic extremes are separated by a narrow strip of only 30 km in width. This combination creates a unique encounter between the two herbal populations, and a strong climatic gradient, resulting in highly-stressful conditions. Thus, the plants from the Judea region have the potential to contain high quantities of various phytochemicals with promising therapeutic properties [2,3,4,5,6,7,8,9]. As a consequence of this permanent stress, both endemic and widely distributed representatives of Mediterranean flora growing in the area have unique chemical contents in plants [4,5,6,7,8,9].

Many shrubs were shown to have various medicinal properties [7]. *Phlomis viscosa* Poiret is an evergreen shrub, who’s areal is limited to Israel, Turkey, Lebanon, and Syria [8]. In fact, *P. viscosa* from the Judea region belongs to a distinct chemotype [9]. We mentioned some compounds of *P. viscosa* [6]. Table 1 summarizes data on compounds identified by us. Our previous publication is devoted to diosmin which is responsible for the anti-inflammatory and anti-diabetic properties of *P. viscosa*.

Chronic wounds affect 6.5 million patients in the United States alone [10]. It is widely acknowledged that the number of chronic wounds is expected to increase worldwide due to the rise in the incidence of age-related conditions and pathologies such as diabetes, obesity, and cardiovascular diseases [1,10]. Unfortunately, less attention, including the phytomedical approach, is paid to this problem than to other major age-related pathologies.

Wound healing (WH) is a complex biological process consisting of a synchronized chain of molecular events aimed at repairing the damaged tissue and restoring its protective barrier function [11]. In general, wound repair occurs in almost all tissues after exposure to any kind of destructive stimulus. This is particularly relevant for the skin—an organ that sustains insult and injury throughout life.

The current therapeutic agents have inadequate efficacy and many serious adverse effects in treating wounds, as suggested by us and others [1,10,12,13]. Anti-platelet agents, aspirin, or non-steroidal anti-inflammatory drugs are the most prescribed at the coagulation phase [14]. Glucocorticoids are also used in many cases since they inhibit the production of hypoxia-inducible factor-1 (HIF-1) [15], but on the other hand, they may promote wound infection [12] and have side effects on the central nervous system [13]. Thus, they should be administered in combination with antibiotics and antiseptics. Some chemotherapeutic drugs are widely used as they inhibit cellular metabolism, rapid cell divisions, and angiogenesis, but on the other hand, they significantly decrease functions of the immune system and often cause excessive bleeding at the wound site [12]. In view of the drawbacks of the current therapies, it is very important to find new therapeutic agents that must be less toxic and more effective than the existing drugs. From this perspective, the medicinal plants may serve as alternative means for advancing WH with fewer side effects. With this in mind, we have examined the pro-WH activity of *P. viscosa*.

While the pro-wound healing (WH) activity of *P. viscosa* has been reported [7], the pro-WH properties of the Israeli chemotype and associated compounds have not been investigated so far in- depth.

Inflammation is one of the most important responses to injury [10] characterized by the involvement of pro-inflammatory cytokines IL-6, IL-8, and so on [7,10,16]. One of the hallmarks of inflammation is the increased secretion of cytokines and the delay in the WH process [7,10,16]. Thus, we examined if the tested extract/compounds may decrease the secretion of pro-inflammatory cytokines.

An additional component of the presented problem is the negative effect of certain types of microorganisms on wound healing [10,11,12,13,14,15,16,17]. The recognition of the fact that antibiotic resistance is one of the major threats during wound healing pushes forward the need in developing novel strategies to overcome microbial acquired resistance towards conventional antibiotics. To the best of our knowledge, no information exists about effective anti-microbial properties of *P. viscosa.*

Thus, the aim of the present study was to investigate pro-WH activities of active phytochemicals of *P. viscosa* and their anti-microbial properties.

## 2. Experimental Section

### 2.1. Preparation of Plant Material

Aerial parts of *P. viscosa* were collected from the Hebron Hills region near Moshav Carmel (Israel). Leaves, flowers, and stems of *P. viscosa* were dried by lyophilization and grounded for Gas chromatography/mass spectrometry (GC/MS) analysis. 

Ethanolic, aquatic, and methanolic extracts were prepared from leaves, stems, and flowers of *P. viscosa* as described by us elsewhere [5]. Since the most effective and the least toxic were ethanolic leave extracts, they were further studied. Plant tissues were homogenized, incubated at room temperature for 48 h in ethanol, centrifuged at 2000 rpm for 10 min, and the supernatant was evaporated by lyophilization. The pellet was dissolved in a minimal amount of 95% ethanol (0.5 mL) and diluted with water to a final concentration of 10 mg/mL. The pelleted plant material was dissolved each time, a new experiment was started to ensure freshness of the stocks.

### 2.2. Identification of Plant Compounds

GC/MS analysis was used for the identification of volatile compounds as previously described by us [6]. GS/MS, a Varian CP 3800 GS/MS analytical system was applied. Headspace injection mode use allowed performing qualitative analysis without extracting active compounds. A modified (data collected and updated for 5 years on the basis of the current experience) analytical library (National Institute of Standards and Technology (NIST) standard reference database) was applied. The probability of compound identification was estimated by comparing its spectrum with those found in the NIST library.

Extracts were separated into different fractions using reverse-phase RP-C18 Sepack column (Supelco, St. Louis, MO, USA) with rising methanol gradient as follows: 0%(v/v), 20%(v/v), 40%(v/v), 60 %(v/v), 80 %(v/v), and 100 %(v/v). An active compound was present in the flavonoid fraction (80%). Identification of diosmin was performed using high-performance liquid chromatography (HPLC), Liquid chromatography-electrospray ionization-mass spectrometry (LC-ESI-MS), and matrix-assisted laser desorption/ionization-time-of-flight-mass spectrometry (MALDI-TOF-MS) comparing with the standard compound. A commercial diosmin with retention times of 23.45 was compared with a pick of flavonoid fraction, which had a similar retention time; an analytical spike test; molecule fragments analysis confirmed the presence of diosmin. NMR results also showed that this compound was diosmin.

### 2.3. Compounds

Diosmin, 1-octen-3-ol and himachala-2,4-diene, were purchased from S.L.Moran, Jerusalem, Israel. The solvents for HPLC tests were obtained from Merck, Kenilworth, N.J., U.S.A. Diosmetin was chemically synthesized as described previously [6].

### 2.4. In Vitro Wound Healing Assay

Estimation of wound healing activity was performed using Human Dermal Fibroblasts (HDF), which were grown in Dulbecco modified Eagle medium (DMEM) containing 10% fetal calf serum, 4 mM of L-Glutamine, and antibiotics under standard conditions of 37 °C and 5% CO_2_. The toxicity of the tested compounds was determined by the neutral red cytotoxicity test [5], observation of morphological changes in cells. HDF cells were placed in 96-well plates, then extracted, and the tested compounds were added 24 h after cell plating. Fibroblast culture without the addition of plant extract or compounds was used as a control. Cells were incubated for an additional 24–72 h, after which the medium was aspirated, and cells were washed with PBS and incubated with a neutral red solution (0.21% in ethanol/water solution 1:100) for 2 h. After washing with PBS, cells were solubilized with Sorenson’s buffer (30.5 mM disodium citrate, 19.4 mM HCl, 50% ethanol) for 15 min with agitation, and the intensity of color was estimated in a plate reader at 570 nm.

After the determination of the non-toxic concentrations of the tested compounds, WH modifying activity was estimated using the scratch assay [7]. In order to carry out the scratch assay, the Ibidi silicone inserts were used (Ibidi, Fitchburg, WI, USA) in accordance with manufacturer recommendations. The inserts were placed into 12-well plates. Experimental procedure included seeding HDF cells on both sides of the inserts, the creation of similar wound gaps after removing the inserts (24 h period incubation), and the addition of the tested compounds in non-toxic concentrations. The rate of gap closure was estimated based on measurements of the cell-free area (mm^2^) as a function of time using a Nikon ELWD microscope (Minato-ku, Tokyo, Japan). The measurements were performed at 0, 10, 20, 24, and 34 h after the addition of the tested compounds.

### 2.5. In Vivo Wound Healing Assay

For the in vivo wound healing assay, we used C57BL/6J male mice. The mice were 4 months old. For each experimental group, five mice were cared for according to the guidelines set forth by the Ben-Gurion University of the Negev Animal Care and use Committee. Ethical animal care permission number IL-65-11-2017 (D). Food and water were supplied ad libitum. The mice were maintained individually during the whole experiment. The experimental design was developed according to the existing WH associated procedure [18,19,20].

Briefly, the animals were anesthetized by intraperitoneal injection using a mixture of ketamine 100 mg/kg and xylazine 10 mg/kg, diluted in 100 µl of saline solution. Hair was removed from the mice dorsum by a shaving machine. The surgical site was treated with 70% alcohol for disinfection. A marker was used to label the spine on the skin. The skin was folded cranially and caudally at midline, according to the label. Then the animal was laid to the lateral position and a 5 mm diameter sterile disposable biopsy punch was used to completely remove all skin layers and create symmetrical full-thickness excisional wounds. These wounds were divided into three equal groups. One group served as control and no pro-WH substances were added to the inflicted wound (sham topical application of non-active cream); while the remaining groups received a topical application of the cream, containing herbal compounds [21]. The wounds were pictured daily, with a ruler attached. After that, the ImageJ software was used to measure the wound area in cm^2^, using the ruler as a reference.

### 2.6. The Constituents of the Creams Were as Follows

1. Control: 10% cetyl alcohol, 7% isopropylmeristat, 21% Vaseline as cream base 1.5% span20, 1.5% tween80 as emulsifying agent, and 0.02% propylparaben as preservative. 15% volume of active cream contained ethanol and distilled water.
2. Diosmin: 10% cetyl alcohol, 7% isopropylmeristat, 21% Vaseline as cream base 1.5% span20, 1.5% tween80 as emulsifying agent, and 0.02% propylparaben as preservative. 15% volume of active cream contained 1 mg diosmin and distilled water.
3. Combination: 10% cetyl alcohol, 7% isopropylmeristat, 21% Vaseline as cream base 1.5% span20, 1.5% tween80 as emulsifying agent, and 0.02% propylparaben as preservative. 15% volume of active cream contained 1 mg diosmin, 0.5 mg 1-octen-3-ol, 0.5 mg himachala-2,4-diene and distilled water.

After the treatment, the animals were monitored until full recovery from anesthesia and returned back to the regular cage. Dipyrone was added to their water, for pain reduction. The wounds were pictured daily, with a ruler attached. After that, the ImageJ software was used to measure the wound area in cm^2^, using the ruler as a reference.

### 2.7. Bacterial Cultures

Lyophilized powders of *Staphylococcus aureus* (ATCC 29213), *Pseudomonas aeruginosa* (ATCC 9027), and *Escherichia coli* (ATCC 25922) were obtained from ATCC collection. The bacteria were re-cultivated using standard buffered peptone water (Acumedia™ 7365A) according to manufacturer instructions. Then the bacteria were inoculated onto specific agar surfaces of: Harlequin™ TBGA (LabM Cat. HAL003) for *Escherichia coli*, cetrimide agar (Sigma-Aldrich Cat 70887-F) for *Pseudomonas aeruginosa*, and mannitol salt agar (Acumedia™ Cat.A7143) for *Staphylococcus aureus*. Colonies of the bacteria were reinoculated into peptone water growing media and incubated at 35 °C for 24 h in the case of *Staphylococcus aureus* and *Escherichia coli*, and 48 h in case of *Pseudomonas aeruginosa*. During cultivation, the cultures were rinsed by centrifugation (6000 RPM) in iso-normal PBS and re-suspended in PBS (5 mL for each type of the bacteria). The microbes were enumerated at specified time points by the Heterotrophic plate counts (HPC) method with pour plate inoculation onto selective agar media. For the experiment, the bacteria were treated with 2 concentrations of the initial extract. The concentrated one was 2.6 mg/mL, while the diluted one was 0.1 mg/mL. Ethanol was used to dissolve the phytochemicals: diosmin, alpha-terpinene and diosmetin; the concentration of each compound was 5 mg/mL.

### 2.8. Pro-Inflammatory Cytokines Level Measurements

Fresh stocks of the extracts and compounds were prepared before each experiment. The concentration of the extracts and compounds was 1 mg/mL. The final ethanol concentration was 0.1%. Senescent HDFs were treated with mentioned plant extracts or compounds for three days. After that, the medium was collected and the concentrations of interleukin-6 (IL-6) and interleukin-8 (IL-8) were measured using ELISA kits according to the protocols of R&D Systems (Minneapolis, MN, USA). Standard curves were generated for each plate to determine sample concentration.

Absorbance was determined using SpectraMax Paradigm multi-mode detection platform (Molecular Devices, Sunnyvale, CA, USA), and data were analyzed using GraphPad Prism software (version 6; GraphPad Software, La Jolla, CA, USA).

### 2.9. Statistical Analysis

Independent experiments were repeated three times. For the in vitro experiments, each one had three replicates. For the in vivo experiments, each group consisted of five mice. All data were analyzed using Statistica for Windows software (StatSoft, Inc., Tulsa, OK, USA), and *p* < 0.05 was chosen as the minimal acceptable level of significance. Simple regression models were subsequently used to eliminate non-significant effects. Values are presented as means ± SD.

## 3. Results

Many active phytochemicals of *P. viscosa* were identified by us previously [6]. The identified compounds are summarized in Table 1.

Incubation of exponentially growing HDF cells with increasing concentrations of the crude extract and pure compounds allowed estimating concentrations, which were acceptable for the WH scratch assay. No cytotoxicity was observed at a concentration below 500 µg/mL for crude extract and diosmin, while the other tested compounds were toxic for the cells at concentrations of more than 100 µg/mL. The most toxic compound was isovaleraldehyde, which caused cell death at a concentration of 10 µg/mL. Of note, cytotoxicity of aldehydes increased with the rise in the number of conjugated double bonds. Remarkably, the combination of diosmin at a concentration of 50 µg/mL with each compound at a concentration of 50 µg/mL (the dose was not cytotoxic for individual compounds) caused a dramatic increase in cell death rates. The mixture of compounds was not toxic for cells when the concentration of diosmin was less than 100 µg/mL and the concentration of other components, except isovaleraldehyde, was less than 20 µg/mL. Also, we have tested the effects of our solvent on the cells. The ethanol at the used concentrations had no effect on the cells.

**Figure 1:**
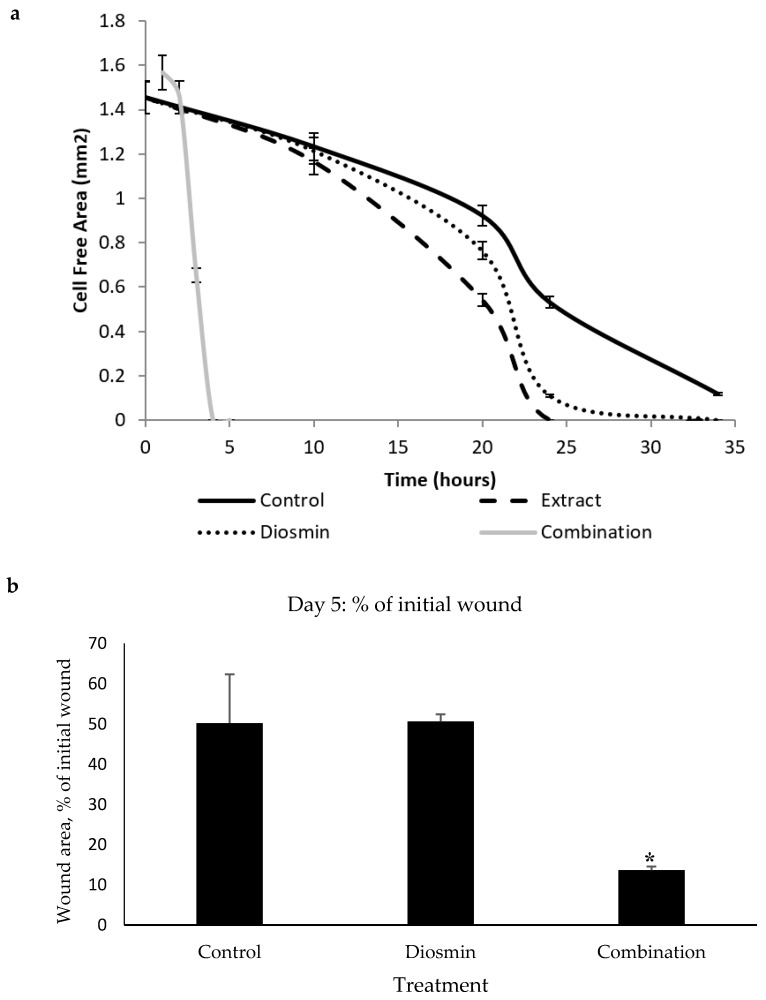
The effect of *P. viscosa* extract and compounds on wound healing. (**a**) The rate of gap closure in cultured human dermal fibroblasts (scratch assay—in vitro model of wound healing) at 0, 10, 20, 24, and 34 h after wound generation. Control was untreated fibroblasts. Crude extract and diosmin were added at a concentration of 50 µg/mL. The combination of tested compounds included diosmin at a concentration of 50 µg/mL, 1-octen-3-ol at a concentration of 10 µg/mL, and himachala-2,4-diene at a concentration of 10 µg/mL. Data from three independent experiments are shown (mean ± SD). *p* < 0.001. (**b**) The effect of diosmin (50 µg/mL) and of the combination diosmin (50 µg/mL), 1-octen-3-ol (10 µg/mL) and himachala-2,4-diene (10 µg/mL) on wound healing process in mice. Full-thickness excisional wounds were lubricated daily with cream, containing diosmin or the combination, described above. The wounds of control mice were lubricated with vehicle cream that contains all compounds except diosmin and the combination. The figure is based on the wound area on day 5. * *p* < 0.05.

The following stage of research included estimation of the pro-WH activity of every identified compound in comparison with the whole extract. Remarkably, only diosmin significantly (*p* < 0.001) accelerated gap closure at a concentration of 50 µg/mL, while other compounds did not have any significant effect. While diosmin had some pro-WH activity, it was less pronounced than that of the crude extract (Figure 1a). Diosmin was tested in various combinations with other identified compounds. Only a combination of three compounds: diosmin, 1-octen-3-ol, and himachala-2,4-diene showed significant pro-WH effect (Figure 1a) as the difference between the control and the combination was pronounced at the late stages of gap closure (*p* < 0.001). In addition, this mixture of compounds was not toxic to cells (unpresented data). After obtaining these data, we have taken the decision to proceed to the in vivo studies. For these studies, we have selected to use only the diosmin and the effective combination of three compounds. The compounds and the combinations that were not effective in the gap closure assay were not tested in mice.

The monitoring of wound closure in mice showed that the cream containing a combination of three compounds: diosmin, 1-octen-3-ol, and himachala-2,4-diene showed the most significant pro-WH effect (*p* < 0.05) on the fifth day of the experiment (Figure 1b) while on other days the effect was less impressive.

**Figure 2:**
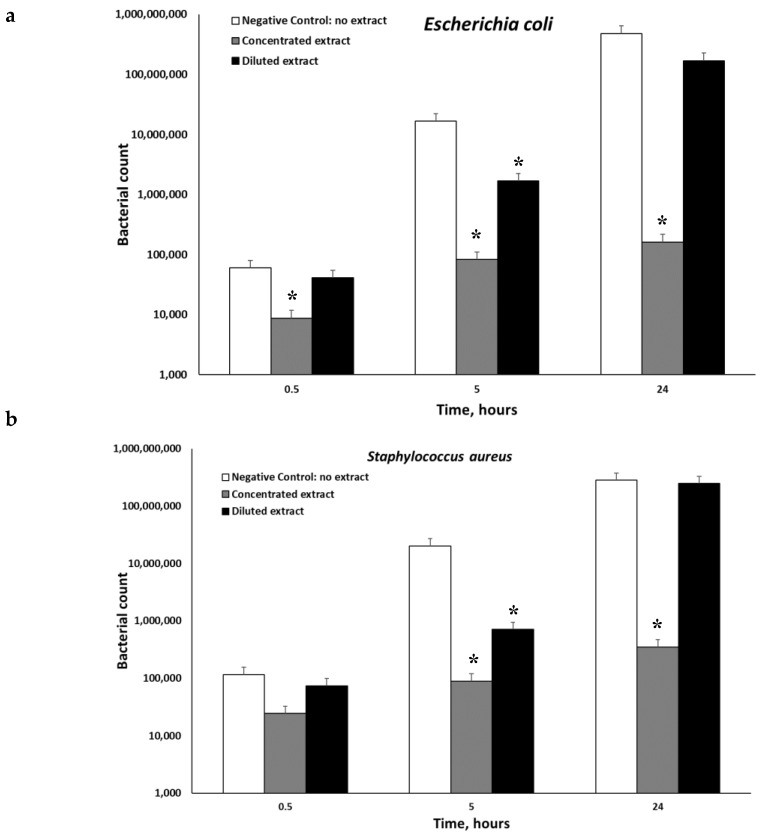

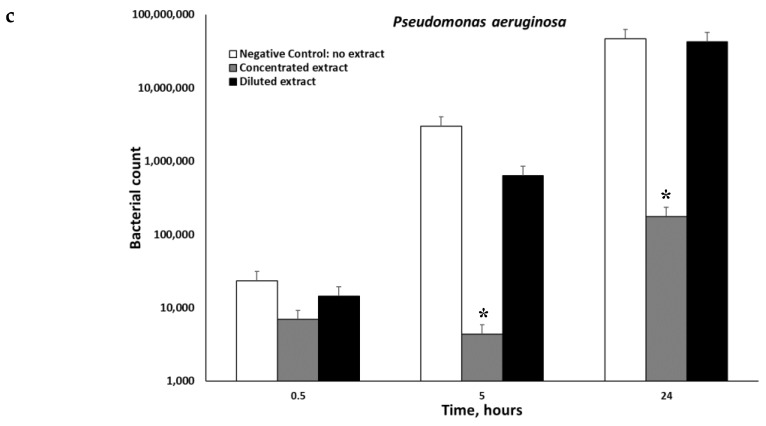
Dynamics of microbial inactivation be *P. viscosa* extract. The concentration of the initial extract was 2.6 mg/mL, while that of the diluted one was 0.1 mg/mL. The antimicrobial activity was examined in vitro against *Escherichia coli* (**a**), *Staphylococcus aureus* (**b**), and *Pseudomonas aeruginosa* (**c**) by using the heterotrophic plate counts method. Data from three independent experiments are shown. (*p* < 0.001). * *p* < 0.05.

**Figure 3:**
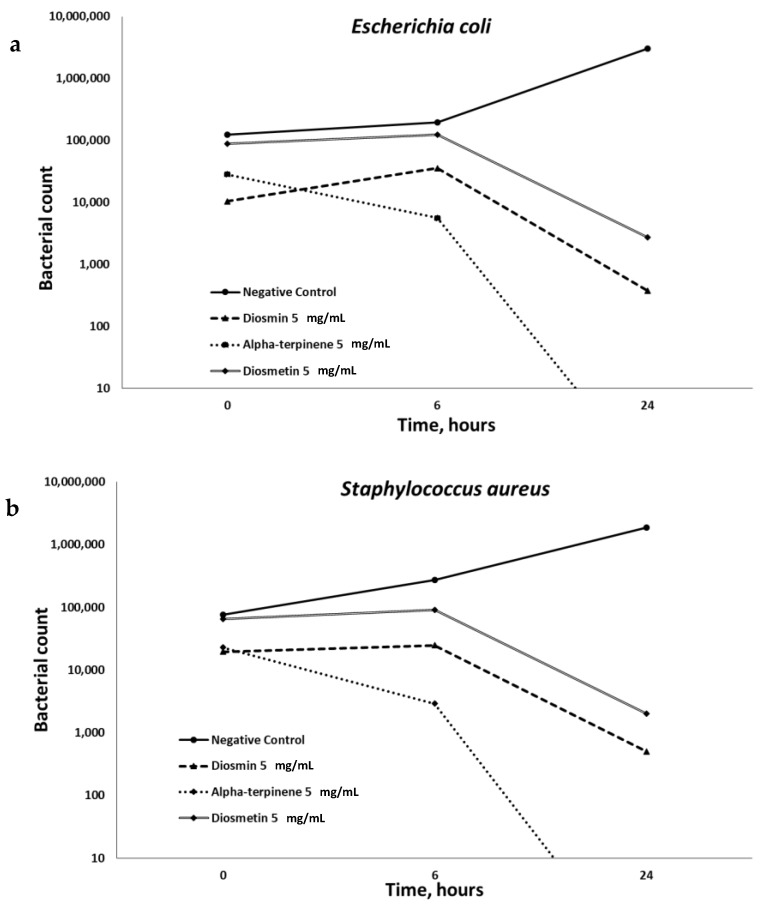

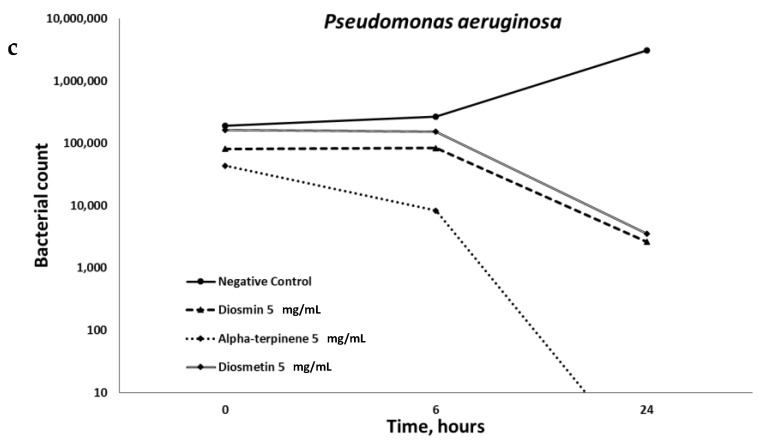
Dynamics of microbial inactivation by Diosmin, Alpha-terpinene, and Diosmetin. The antimicrobial activity of different compounds was examined in vitro against *Escherichia coli* (**a**), *Staphylococcus aureus* (**b**), and *Pseudomonas aeruginosa* (**c**), by using the heterotrophic plate counts method. Data from three independent experiments are shown, (*p* < 0.001).

The anti-microbial properties of the extract were investigated. Three different microorganisms *Escherichia coli*, *Staphylococcus aureus*, and *Pseudomonas aeruginosa* were tested. Figure 2 demonstrates that the extracts of *P. viscosa* inhibited microbial growth in all cases, but significantly only (*p* < 0.001) when the concentrated extracts were used. Both diluted and concentrated extracts had significant anti-microbial activities against *Escherichia coli* (Figure 2). Figure 3 shows that the best anti-microbial properties belong to alpha-terpinene.

**Figure 4:**
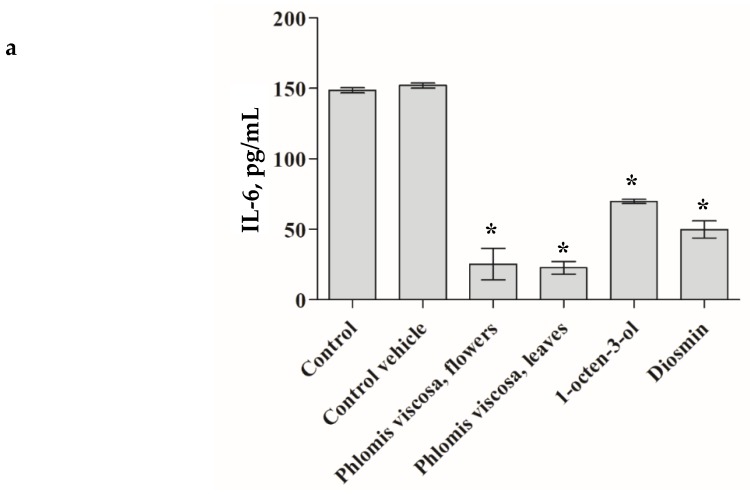

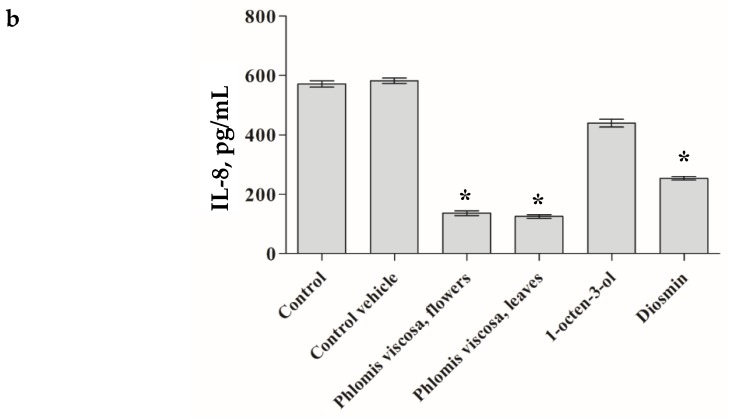
The anti-inflammatory effect of *Phlomis viscosa* extracts and compounds. The effect of *Phlomis viscosa* extracts and compounds on the secretion of pro-inflammatory cytokines, IL-6 (**a**) and IL-8 (**b**) by human dermal fibroblasts. The concentration of the extracts and compounds was 1 mg/mL. The final ethanol concentration was 0.1%. Untreated cells were used as controls. Cells treated with 0.1% ethanol (Control vehicle) were used to exclude the effect of ethanol on the cells. Data from three independent experiments are shown, (*p* < 0.001). * *p* < 0.05.

The *P. viscosa* extracts and its identified phytochemicals were investigated from the perspective of chronic inflammation. As seen in Figure 4, only four items (*P. viscosa* extract of flowers, that of leaves, diosmin, and 1-octen-3-ol) significantly decreased secretion of IL-6 and IL-8 cytokines by senescent HDFs as measured by ELISA assay (*p* < 0.001).

## 4. Discussion

We identified the presence of diosmin in the crude extract of *P. viscosa* [6]. Given that oral or topical administration of micronized flavonoid fraction that included 90% of diosmin in the infected wounds was effective in an animal model [22], it was logical to assume it as the main pro-WH component of the extract. In fact, many studies focused on the role of diosmin in the healing of venous ulcers [23,24,25,26], resulting in the development of the drug Daflon which is based on a purified flavonoid fraction containing 90% diosmin [24]. In general, beneficial properties of diosmin were most pronounced in combination with other compounds. This is evident from synergistic diosmin-based formulations that were effective in treating venous insufficiency, hemorrhoids, lymphedema, diabetes, melanoma, dermatitis, mastalgia, colitis, and pre-menstrual syndrome [27]. With this in mind, we have tested various combinations of the identified compounds with diosmin. Surprisingly, the double combination of diosmin with each tested compound did not show a strong pro-WH effect, as only the combination of the three above-mentioned compounds was the most potent (Figure 1).

We tested the pro-inflammatory cytokines IL-6 and IL-8 expecting that the extract and some compounds of the extract may decrease secretion of these pro-inflammatory cytokines taking into account that namely the above-mentioned cytokines clearly reflect a delay in the WH process and are known to be secreted by HPF [7,9,10,16]. Figure 4 demonstrates that the extracts from leaves and flowers of the tested plant and its two compounds (diosmin and 1-octen-3-ol) significantly (*p* < 0.001) decreased secretion of the pro-inflammatory cytokines.

The molecular mechanism behind WH acceleration by the identified phytochemicals combination remains to be investigated. Taking into account complex mechanisms underlying the process of WH and lack of information regarding the contribution of diosmin to this process, it is still unclear how exactly diosmin participate in the WH process and how all components synergize in it. Nevertheless, it was reported that in case of ethanol-induced hepatic injury diosmin managed to modulate inflammation by alleviating ethanol-induced NF-κB (nuclear factor kappa-light-chain-enhancer of activated B cells) activation and enhancing expression of TNF-α (tumor necrosis factor alpha), COX-2 (Cyclooxygenase-2) and iNOS (inhibitor, nitric oxide synthase) [28]. 1-octen-3-ol and himachala-2,4-diene, and other components of the combination, were never reported, to the best of our knowledge, as compounds capable of enhancing WH. Only anti-inflammatory activity of edible mushroom containing 1-octen-3-ol was mentioned [29]. Interestingly, our report on the anti-inflammatory activity of 1-octen-3-ol, that is decrease in the secretion of IL-6 and IL-8, is in agreement with the results obtained by Chen et al. [29].

An additional challenge to successful WH resolution is the contamination of the wounds with certain types of microorganisms: the most widespread of which are *Escherichia coli*, *Staphylococcus aureus*, and *Pseudomonas aeruginosa* [16]. The compounds involved in the anti-microbial action of the tested extract are alpha-terpinene and diosmin (Figure 3). To the best of our knowledge, the anti-microbial properties of these compounds were never mentioned. Bacterial quorum sensing is a form of regulatory, cell-to-cell communication and associated with many bacterial properties including genetic competence, bacterial colonization, biofilm formation, virulence and so on, which makes many bacterial phenotypes more dangerous [30]. This phenomenon primarily affects chronic wounds containing bacterial biofilms. It was demonstrated that 60% of chronic wounds had bacterial biofilms, as opposed to only 8% of acute wounds [31]. Further studies are necessary in order to determine the mechanism of bacterial biofilms’ influence on WH and to identify bioactive compounds capable of disrupting biofilms, produced by infectious bacteria. The role of quorum sensing in delayed healing of different wounds remains to be elucidated. Thus, studies on medicinal plants with identified quorum sensing inhibitory activity are required.

Previously, we demonstrated the anti-inflammatory effects of Diosmin as well as enhancing the effects of Diosmin on glucose uptake of the adipocytes [6]. Given the deep links between complex poly-genetic human diseases, especially those with age component [32,33], plant materials are already known to be effective against at least one of these diseases are more likely to be active against the others. This could be clearly seen from the properties of diosmin.

Collectively, our results suggest a mechanistic basis for the potential use of the identified plant phytochemicals combination in the treatment of chronic wounds. Finally, the elucidation of the mechanisms of action of the tested compounds might aid in improving the design of new pro-WH drugs.

## Figures and Tables

**Table 1:** Identification of major compounds of *P. viscosa.*

Compound	Plant Organ	Probability of Compound Identification (%)
Diosmin	Leaves	98.7
Quercetin 3-O-rutinoside	Leaves, flowers	96.4
Isovaleraldehyde	Leaves, flowers, stems	84.0
2,4-hexadienal	Leaves	83.5
2-hexenal	Leaves	87.2
alpha-terpinene	Leaves	86.5
1-octen-3-ol	Leaves	87.3
himachala-2,4-diene	Leaves, flowers	85.3
N-octanal	Flowers	87.3
Bourbonene	Flowers	89.2
1-propanal, 2-methyl	Stems	89.4
Cubebene	Leaves, flowers	87.3

## References

[1] Budovsky A., Fraifeld V.E. (2012). Medicinal plants growing in the Judea region network approach for searching potential therapeutic targets. Netw. Biol..

[2] Lev E., Amar Z. (2000). Ethnopharmacological survey of traditional drugs sold in Israel at the end of the 20th century. J. Ethnopharmacol..

[3] Gorelick J., Kitron A., Pen S., Rosenzweig T., Madar Z. (2011). Anti-diabetic activity of Chiliadenus iphionoides. J. Ethnopharmacol..

[4] Tamir H., Satovic Z., Gorelick J., Danin A., Fischer R., Chaimovitsh D., Dudai N. (2011). Intraspecific variation of Chiliadenus iphionoides essential oil in Israel. Chem. Biodivers..

[5] Yarmolinsky L., Budovsky A., Danilenko M., Maor H., Wolfson M., Fraifeld V., Ben-Shabat S. (2015). Anti-cancer properties of Varthemia iphionoides (Chiliadenus iphinoides). Isr. J. Plant Sci..

[6] Yarmolinsky L., Budovsky A., Ben-Shabat S., Khalfin B., Gorelick J., Bishitz Y., Miloslavski R., Yarmolinsky L. (2018). Recent updates on the phytochemistry and pharmacological properties of *Phlomis viscosa* Poiret. Rejuvenation Res..

[7] Altemimi A., Lakhssassi N., Baharlouei A., Watson D.G., Lightfoot D.A. (2017). Phytochemicals: Extraction, Isolation, and Identification of Bioactive Compounds from Plant Extracts. Plants.

[8] Agyare C., Boakye Y.D., Bekoe E.O., Hensel A., Dapaah S.O., Appiah T. (2016). Review: African medicinal plants with wound healing properties. J. Ethnopharmacol..

[9] Budovsky A., Shteinberg A., Maor H., Duman O., Yanai H., Wolfson M., Fraifeld V., Shteinberg A., Maor H., Duman O. (2014). Uncovering the Geroprotective Potential of Medicinal Plants from the Judea Region (Israel). Rejuvenation Res..

[10] Budovsky A., Yarmolinsky L., Ben-Shabat S. (2015). Effect of medicinal plants on wound healing. Wound Repair Regen..

[11] Gurtner G.C., Werner S., Barrandon Y., Longaker M.T. (2008). Wound repair and regeneration. Nature.

[12] Guo S., Dipietro L.A. (2010). Factors affecting wound healing. J. Dent. Res..

[13] Ciriaco M., Ventrice P., Russo G., Scicchitano M., Mazzitello G., Scicchitano F., Russo EVentrice P. (2013). Corticosteroid-related central nervous system side effects. J. Pharmacol. Pharmacother..

[14] Giannobile W.V., Somerman M.J. (2003). Growth and amelogenin-like factors in periodontal wound healing. A systematic review. Ann. Periodontol..

[15] Wagner A.E., Huck G., Stiehi D.P., Jelkmann W., Hellwig-Burkel T. (2008). Dexamethasone impairs hypoxia-inducible factor-1 function. Biochem. Biophys. Res. Commun..

[16] Bowler P.G., Duerden B.I., Armstrong D.G. (2001). Wound microbiology and associated approaches to wound management. Clin. Microb. Rev..

[17] Kadam S., Shai S., Shahane A., Kaushik K.S. (2019). Recent Advances in Non-Conventional Antimicrobial Approaches for Chronic Wound Biofilms: Have We Found the ‘Chink in the Armor’?. Biomedicines.

[18] Seidman R., Gitelman I., Sagi O., Horwitz S.B., Wolfson M. (2001). The role of ERK 1/2 and p38 MAP-kinase pathways in taxol-induced apoptosis in human ovarian carcinoma cells. Exp. Cell Res..

[19] Moreira C.F., Cassini-Vieira P., da Silva M.F., Barcelos L.S. (2015). Skin Wound Healing Model—Excisional Wounding and Assessment of Lesion Area. Bio-Protocol.

[20] Wu Y.C., Kulbatski I., Medeiros P.J., Maeda A., Bu J., Xu L., Chen Y., DaCosta R.S. (2014). Autofluorescence imaging device for real-time detection and tracking of pathogenic bacteria in a mouse skin wound model: Preclinical feasibility studies. J. Biomed Opt..

[21] Namjoyan F., Kiashi F., Moosavi Z.B., Saffari F., Makhmalzadeh B.S. (2015). Efficacy of Dragon’s blood cream on wound healing: A randomized, double-blind, placebo-controlled clinical trial. J. Tradit. Complementary Med..

[22] Hasanoglu A., Ara C., Ozen S., Kali K., Senol M., Ertas E. (2001). Efficacy of Micronized Flavonoid Fraction in Healing of Clean and Infected Wounds. Int. J. Angiol..

[23] Coleridge-Smith P., Lok C., Ramelet A.A. (2005). Venous leg ulcer: A meta-analysis of adjunctive therapy with micronized purified flavonoid fraction. Eur. J. Vasc Endovasc. Surg..

[24] Smith P.C. (2005). Daflon 500 mg and venous leg ulcer: New results from a meta-analysis. Angiology.

[25] Serra R., Grande R., Butrico L., Buffone G., Caliò F.G., Squillace A., Rizzo B.A., Massara M., Spinelli F., Ferrarese A.G. (2016). Effects of a new nutraceutical substance on clinical and molecular parameters in patients with chronic venous ulceration. Int. Wound J..

[26] González Ochoa A. (2017). Sulodexide and phlebotonics in the treatment of venous ulcer. Int. Angiol..

[27] Szymański M., Młynarek D., Szymański A., Matławska I. (2016). Simultaneous Determination of Diosmin and Hesperidin in Pharmaceuticals by RPLC using Ionic Liquids as Mobile Phase Modifiers. Iran J. Pharm. Res..

[28] Tahir M., Rehman M.U., Lateef A., Khan R., Khan A.Q., Qamar W., Ali F., O’Hamiza O., Sultana S. (2013). Diosmin protects against ethanol-induced hepatic injury via alleviation of inflammation and regulation of TNF-α and NF-κB activation. Alcohol.

[29] Chen C.Y., Chien S.C., Tsao N.W., Lai C.S., Wang Y.Y., Hsiao W.W., Chu F.H., Kuo Y.H., Wang S.Y. (2016). Metabolite Profiling and Comparison of Bioactivity in Antrodia cinnamomea and Antrodia salmonea Fruiting Bodies. Planta Med..

[30] Yarmolinsky L., Bronstein M., Gorelick J. (2015). Review: Inhibition of bacterial quorum sensing by plant extracts. Isr. J. Plant Sci..

[31] James G.A., Swogger E., Wolcott R., Pulcini E.D., Secor P., Sestrich J., Costerton J.W., Stewart P.S. (2008). Biofilms in chronic wounds. Wound Repair Regen..

[32] Tacutu R., Budovsky A., Fraifeld V.E. (2010). The NetAge database: A compendium of networks for longevity, age-related diseases and associated processes. Biogerontology.

[33] Gobshtis N., Ben-Shabat S., Fride E. (2007). Antidepressant-induced undesirable weight gain: Prevention with rimonabant without interference with behavioral effectiveness. Eur. J. Pharmacol..

